# Understanding the modifiable health systems barriers to hypertension management in Malaysia: a multi-method health systems appraisal approach

**DOI:** 10.1186/s12913-015-0916-y

**Published:** 2015-07-03

**Authors:** Isabelle Risso-Gill, Dina Balabanova, Fadhlina Majid, Kien Keat Ng, Khalid Yusoff, Feisul Mustapha, Charlotte Kuhlbrandt, Robby Nieuwlaat, J.-D. Schwalm, Tara McCready, Koon K. Teo, Salim Yusuf, Martin McKee

**Affiliations:** London School of Hygiene and Tropical Medicine, 15-17 Tavistock Place, London, WC1H 9SH UK; Universiti Teknologi MARA, Kuala Lumpur, Malaysia; National Defence University of Malaysia, Kuala Lumpur, Malaysia; UCSI University, Kuala Lumpur, Malaysia; Population Health Research Institute, McMaster University, Hamilton Ontario, Canada

## Abstract

**Background:**

The growing burden of non-communicable diseases in middle-income countries demands models of care that are appropriate to local contexts and acceptable to patients in order to be effective. We describe a multi-method health system appraisal to inform the design of an intervention that will be used in a cluster randomized controlled trial to improve hypertension control in Malaysia.

**Methods:**

A health systems appraisal was undertaken in the capital, Kuala Lumpur, and poorer-resourced rural sites in Peninsular Malaysia and Sabah. Building on two systematic reviews of barriers to hypertension control, a conceptual framework was developed that guided analysis of survey data, documentary review and semi-structured interviews with key informants, health professionals and patients. The analysis followed the patients as they move through the health system, exploring the main modifiable system-level barriers to effective hypertension management, and seeking to explain obstacles to improved access and health outcomes.

**Results:**

The study highlighted the need for the proposed intervention to take account of how Malaysian patients seek treatment in both the public and private sectors, and from western and various traditional practitioners, with many patients choosing to seek care across different services. Patients typically choose private care if they can afford to, while others attend heavily subsidised public clinics. Public hypertension clinics are often overwhelmed by numbers of patients attending, so health workers have little time to engage effectively with patients. Treatment adherence is poor, with a widespread belief, stemming from concepts of traditional medicine, that hypertension is a transient disturbance rather than a permanent asymptomatic condition. Drug supplies can be erratic in rural areas. Hypertension awareness and education material are limited, and what exist are poorly developed and ineffective.

**Conclusion:**

Despite having a relatively well funded health system offering good access to care, Malaysia's health system still has significant barriers to effective hypertension management.

**Discussion:**

The study uncovered major patient-related barriers to the detection and control of hypertension which will have an impact on the design and implementation of any hypertension intervention. Appropriate models of care must take account of the patient modifiable health systems barriers if they are to have any realistic chance of success; these findings are relevant to many countries seeking to effectively control hypertension despite resource constraints.

## Background

The importance of developing robust responses to the growing burden of non-communicable disease (NCD) has been highlighted by two recent events, the 2010 update of the Global Burden of Disease demonstrating the scale of the epidemiological transition to NCDs [1] and the 2011 United Nations High-Level Meeting calling for urgent action to tackle NCDs [2].

Cardiovascular diseases are the largest contributor to the NCD burden worldwide, with hypertension among the most important risk factors [3, 4]. Appropriate responses span public health measures to prevent the onset of disease and health care to tackle risk factors and established disease.

The means to manage hypertension have been available for decades and are cost-effective, requiring no more than a sphygmomanometer for diagnosis, one, or more often a combination, of cheap and effective drugs for treatment [4, 5], but also, and somewhat more difficult to achieve, a health system that can make them available to those in need. The achievable health benefits are large as exemplified by the rapid declines in stroke-related mortality that have occurred in most high income countries during the past four decades [6]. Yet these gains have been elusive in many low and middle income countries, with most hypertensive individuals unaware of their risk and, even if they are aware, unable to receive treatment or achieve blood pressure control [7].

Effectively addressing hypertension requires strategies that are multifaceted, involving preventive actions and improved control, while overcoming established structural and institutional barriers. This can appear unachievable in situations with scarce resources and competing priorities, risking a sense of therapeutic nihilism [8]. Consequently, there has been limited attention from researchers and their funders to the potential to deliver a pragmatic response to hypertension in low and middle income country settings.

NCDs are rising rapidly in many low and middle income countries (LMIC), and prevention and control has potential to make large inroads. One such country is Malaysia, an upper middle income country, where we have studied the barriers to effective hypertension detection, treatment and control. Lessons learnt from Malaysia can be applied to many other countries. In the 2011 National Health and Morbidity Survey conducted in Malaysia, which included 28,000 subjects, 32.7 % of participants, aged 18 and over, had hypertension (systolic ≥140 mmHg, and/or diastolic ≥90 mmHg or self-reported on treatment), of whom only 39.1 % were aware of their status [9]; this places Malaysia in the middle of the range of countries in the Western Pacific region of WHO [10]. An earlier nationwide study of over 16,000 subjects, reported in 2007, found 27.8 % of those aged over 15 had hypertension. Only 34.6 % of the subjects with hypertension were aware of their hypertensive status, and 32.4 % were taking antihypertensive medication. Amongst the latter group, only 26.8 % had their blood pressure under control [11]; thus overall only 9.3 % of those with hypertension were controlled and, to the extent that the surveys were comparable, this suggests that the situation may be deteriorating.

In contrast, some facility-based studies have found higher rates of control but they only include patients who are aware of their condition and are able to receive care, thereby suffering from selection bias. For example, one study of nine clinics found that, overall, 59 % of 1260 patients with hypertension were controlled [12]. However, rates varied substantially among clinics from 23.1 to 74.2 %, also indicating the scope for improvement using practices already in place in some settings that can be replicated elsewhere.

The components of a comprehensive health system response are largely agreed. They include diagnosis, appropriate lifestyle and pharmacological treatment, and regular monitoring to achieve control. However, it is far from obvious that models of care developed in Western countries can simply be transplanted to Malaysia as they may be dependent on availability of resources, healthcare system design, cultural influences and other factors.

One extension of the international PURE (Prospective Urban and Rural Epidemiology) study, which is now following up over 180,000 people aged 35 and over in 21 countries to track trends in cardiovascular disease and its risk factors [13], is the HOPE-4 randomized controlled trial. HOPE-4 seeks to design and evaluate an intervention that will comprise fixed single pill fixed dose cardiovascular combination therapy, coupled with advice on self-management, administered by mid-level non physician health workers. However, as with any complex intervention, the implementation will need to take account of the characteristics of the health systems, population preferences and local context. This requires knowledge of the barriers that are likely to arise and how these can be countered.

In this paper we describe the use of a multi-method health system assessment to understand the barriers and facilitating factors for optimal hypertension management (diagnosis, treatment and control) in Malaysia from a patient perspective. This approach has been previously used to assess health systems performance, whereby it uses a specific condition (such as hypertension) as a tracer that can explore the functioning of the diverse elements of the health care system [14]. In this case we use it to identify barriers to effective management that must be addressed in designing an intervention to be evaluated in a subsequent cluster randomized controlled trial.

## Methods

### Theoretical framework

**Fig. 1:**
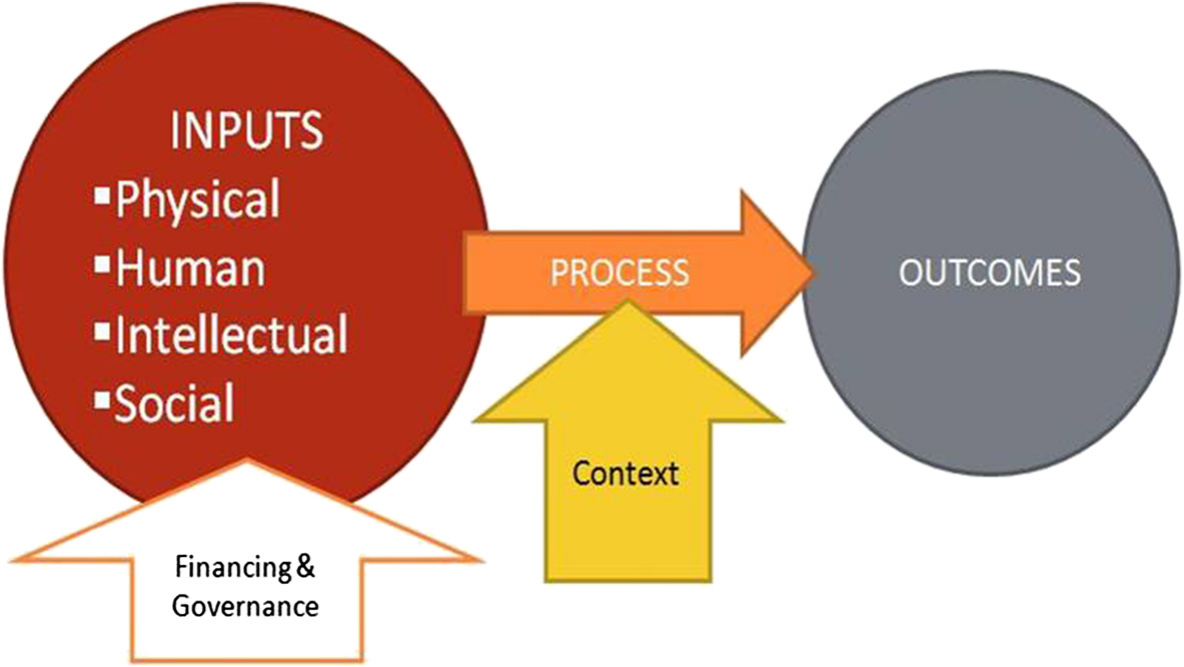
Conceptual framework of health systems assessment

The approach to health systems assessment is underpinned by a conceptual framework (Fig. 1) that draws on the model of health systems proposed in the 2000 World Health Report [15]. Recognizing the criticism that the building blocks approach is somewhat mechanistic evaluating each block separately [16], the study framework is modified to enable an analysis of the relationship between the various resources and components of a health system (Fig. 1). It is informed by the “systems thinking” approach [17] that addresses the complexity of the health system and the interactions of sub-systems leading to changes in the whole. The approach seeks to explain the causes for certain phenomena, and to identify plausible pathways about how resource inputs translate into outcomes. It draws on realistic evaluation [18], in which “mechanism + context = outcome” and where the focus is more on what intervention or policy works in what circumstances. What “works” is assessed from the perspective of the patients and frontline providers, as the ultimate beneficiaries of a well-functioning system.

This multi-method approach is suited to many other complex chronic diseases and has most often been used with diabetes [19–21]. It recognizes that adequate health care funding is necessary but not sufficient for optimal health outcomes, which require the effective management of four types of resources: physical, such as health facilities, medical equipment, and drugs; human, such as trained health workers; intellectual, such as evidence-based guidelines and understanding by patients of the nature of the disease and its management; and social, such as means for patients to afford and access necessary treatment. The framework also takes into account the broader societal context and the overarching roles of governance and finance in determining how the different inputs are transformed into outcomes.

### Document review and synthesis

The first step involved review and synthesis of official statistics, published and grey literature, survey data and government reports to understand the formal health system and how it is expected to work. This involved searches around key framework domains using Google and PubMed, using the terms Malaysia and hypertension and its synonyms, a search of Malaysian government and the World Health Organization websites, documents identified by key informants, and a follow up of references cited.

### Community sampling

This was used to frame the primary research which sought to understand what happens in practice. Study sites were selected from among those participating in the PURE study [22] to cover diverse settings within Malaysia: two urban sites close to the capital, Kuala Lumpur (Petaling Bahagia and Sungai Buloh) and two rural sites, one in Peninsular Malaysia (Tanjung Sepat) and one in rural Sabah (Kota Marudu). The sites were chosen on the basis of significant variations in hypertension prevalence and control, with marked differences not only between urban and rural sites, but also within each type of setting (Table 1). This approach made it possible to capture a diversity of setting-specific barriers.

**Table 1:** Hypertension data from study sites in subjects aged 35–70 (2013)

Community name	N	Location	Diabetes mellitus prevalence	Hypertension prevalence	Of which aware	Of which treated	Of which controlled
Petaling Bahagia	34	Urban	5.9 %	38.2 %	53.8 %	53.8 %	38.5 %
Shah Alam	133	Urban	10.5 %	45.9 %	58.9 %	58.9 %	17.6 %
Tanjong Sepat	127	Rural	7.9 %	57.5 %	46.6 %	32.9 %	9.6 %
Kota Marudu	334	Rural	3.9 %	47.6 %	59.1 %	48.4 %	23.9 %

Field work was undertaken by local researchers, most already working on the PURE project, who received training in health systems appraisal at a workshop held in Kuala Lumpur, using a detailed manual developed for hypertension that contained definitions, sampling frameworks, and assessment tools, seeking to improve consistency across sites. At each site, researchers identified the main public health facilities at which people seek treatment for hypertension, and then identified private and traditional providers serving the community.

### Fieldwork and data analysis

Semi-structured interviews were conducted with a purposive sample of patients with hypertension and health professionals involved in hypertension management (Table 2). Each clinic was visited over the course of a few days. Attendance lists were used as a sampling frame, from which patients being treated for hypertension were selected purposively, with the intention of obtaining a mix of subjects by gender, ethnicity, and hypertension control. Inevitably, those included reflected the population served. Thus, all interviewees from indigenous groups were recruited in the community in Sabah. The interview guides drew on earlier research involving the authors on barriers to diabetes management [19–21] and two systematic reviews conducted to identify health system and service barriers to hypertension control [23, 24]. A convenience sample was used for health professionals, but based on a pre-specified list of those who would have direct patient contact as set out in the study protocol. The interviews, which typically lasted 30 minutes, explored patient pathways (from initial diagnosis to consistent treatment), access to medicines, adherence to treatment, and management of complications. Key informants at national level were identified through documentary analysis and were interviews, including civil servants and representatives of professional organizations, to gain insights into the hypertension-related regulation and policy context. The source of each quotation cited is indicated as key informant (KI), healthcare provider (HP) and patient (PA).

**Table 2:** Characteristics of respondents by site

			Petaling Bahagia (urban, KL)		Tanjung Sepat (rural, peninsula)		Marudu (rural Sabah island)		UiTM (urban, KL)		Total
			M	F	M	F	M	F	M	F	
Type of Informant	Key Informant										12
	Health professional	Public	0	3	1	3	2	3	0	1	13
		Private	2	1	0	0	0	0	0	3	6
		TCM	2	0	2	0	0	0	0	1	5
		Total	8		6		5		5		24
	Patient	Public	2	4	0	2	3	8	6	4	29
		Private	1	0	1	1	0	0	1	1	5
		TCM	0	0	1	0	0	0	0	2	3
		Total	7		5		11		14		37
Patient demographics	Hypertension status	Controlled	3	4	1	3	1	3	7	5	27
		Uncontrolled	0	0	1	0	2	5	0	2	10
	Ethnicity	Malay	1	3	2	3	0	0	7	5	21
		Indian	1	0	0	0	0	0	0	1	2
		Chinese	1	1	0	0	0	0	0	1	3
		Other minority	0	0	0	0	3	8	0	0	11
	Total		15		11		16		19		73

Ethical approval was granted by the respective committees at the Universiti Teknologi MARA and the London School of Hygiene and Tropical Medicine. Following informed consent, interviews were conducted in Bahasa Malaysia or English and recorded. Transcripts were coded inductively using NVivo software eliciting major themes and these were then mapped onto the conceptual framework, constructing a narrative summary. Findings and inferences emerging from the interviews were triangulated with routine statistics, policy documents, published and unpublished literature throughout all stages of the study and data interpretation [25].

## Results

This section reports thematically the barriers and facilitators to effective hypertension management according to the conceptual framework beginning with a description of the management of hypertension. Findings are interpreted in the context of the Malaysian health system (Appendix Tables 4 and 5).

### The patient pathway

**Fig. 2:**
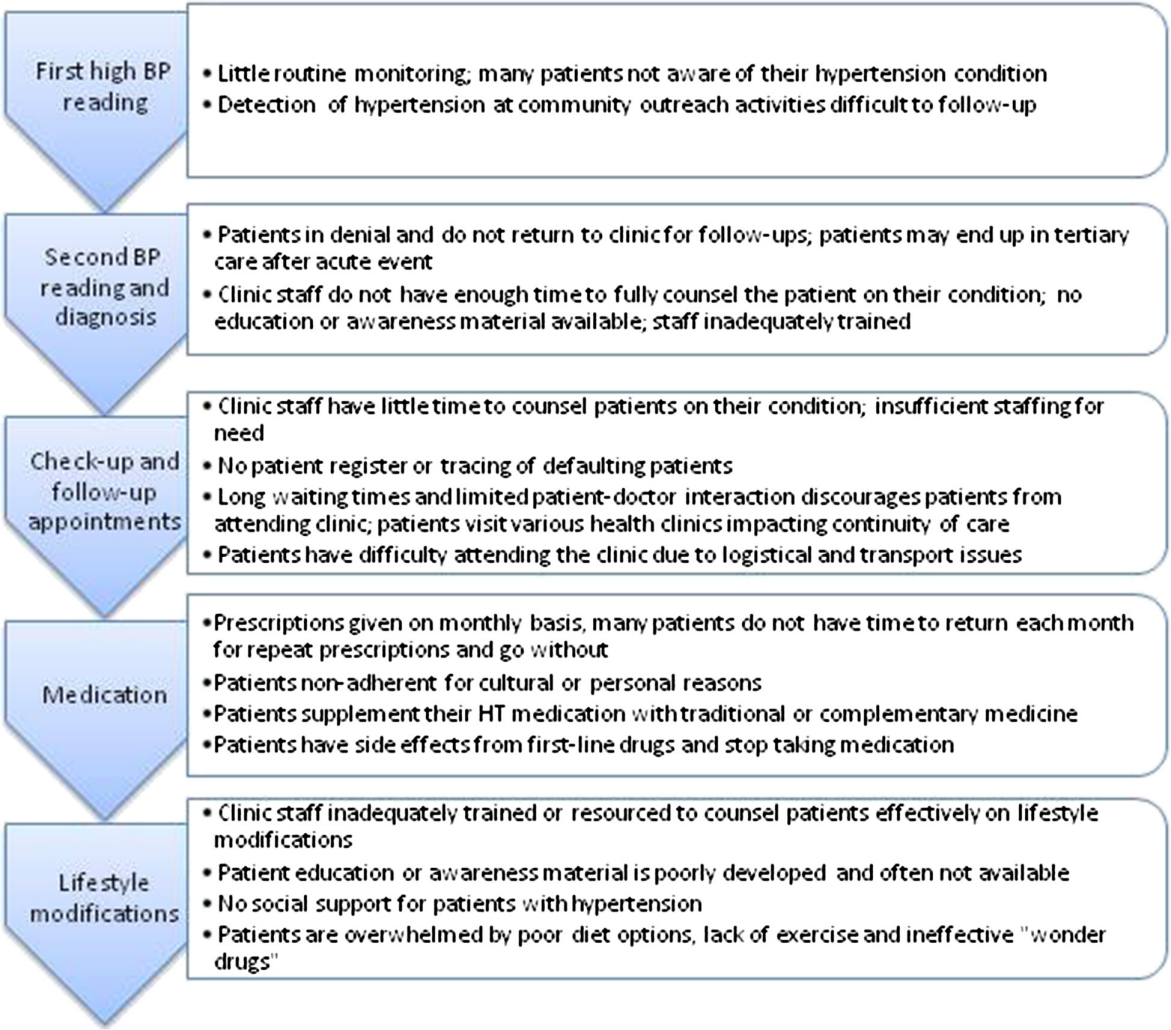
Barriers along hypertension patient pathway

In theory, the patient should follow a standardized pathway [26], with guidelines developed jointly by the Ministry of Health, Malaysian Society of Hypertension, and Academy of Medicine of Malaysia [27]. The guidelines envisage that all clinics should manage patients with hypertension, with staff undergoing specific training in screening and hypertension management. Fig. 2 illustrates the barriers to effective hypertension care along the patient pathway.

#### Screening and diagnosis

Most patients with hypertension reported being diagnosed after presenting to a clinic with symptoms, although none of those interviewed suspected that their symptoms might indicate hypertension. Blood pressure is not routinely checked during attendance at primary care clinics for other problems, contrary to national guidelines; however some doctors do measure BP in all patients visiting the clinics. Some clinics also have “self-checking corners” with a digital sphygmomanometer, to *“encourage or empower the public to look after themselves”* (KI) although there are no data on their utilization. Very few asymptomatic patients ask for their BP to be checked, with one Medical Officer noting: *“Rarely people came in just to have their BP checked [without symptoms], because they think they are still young so why they need to have a health check-up?”*

Mass screening does, however, occur regularly, both at work and in health promotion sessions (typically combined with blood donation) known as “Family Days”, undertaken by some clinics in the local community, usually in markets or outside mosques. One physician in an urban clinic reported that *“we usually detect 30 % asymptomatic undiagnosed hypertension during our health campaigns”*. However, few of those identified with elevated blood pressure return for confirmation of the diagnosis even though they appear concerned about the initial reading.

#### Follow-up

The guidelines recommend that those with controlled hypertension receive check-ups every 4–6 weeks until they are controlled and then every 3–6 months. Patients report that this largely happens. In the public sector, a nurse will take the patient’s blood pressure readings and any other tests required, which are then followed up by the Medical Officer as the nurse is not allowed to prescribe. Commonly the Medical Officer will just *“tell the patient to continue medication, sometimes without physical examination”* (HP); they report having little time to talk with patients, and they simply *“take their [the patient’s] word”* as to whether they are adhering to medication and modifying their lifestyle as the doctors have insufficient time to engage with them to ensure a shared understanding. If a patient’s blood pressure continues to be uncontrolled or they have complications, the Medical Officer will refer them to a Family Medical Specialist (FMS), a physician with advanced training in primary care, for further investigation and management. Physicians in the public sector reported how some clinics struggle to cope with their workload. Some clinics offer dedicated sessions for hypertension and its complications on specified days of the week, designed to allow more time for detailed assessments and patient education. However, physicians reported seeing 10 or more patients per hour, or 100 in a day, leaving inadequate time for meaningful interaction: *“Many of healthcare providers [are] not able to sit down and have counselling session regarding their medications with their patients”* (KI). Other informants speculated that pharmacists and nutritionists could also play a larger role in patient care although they are not always present in facilities. Patients face substantial waiting times. At one clinic only half those scheduled to attend actually did, most of whom had another condition, such as diabetes. In the private sector the physicians will spend more time not only taking the patient’s blood pressure but advising on medication and lifestyles.

#### Non-adherence to treatment

**Fig. 3:**
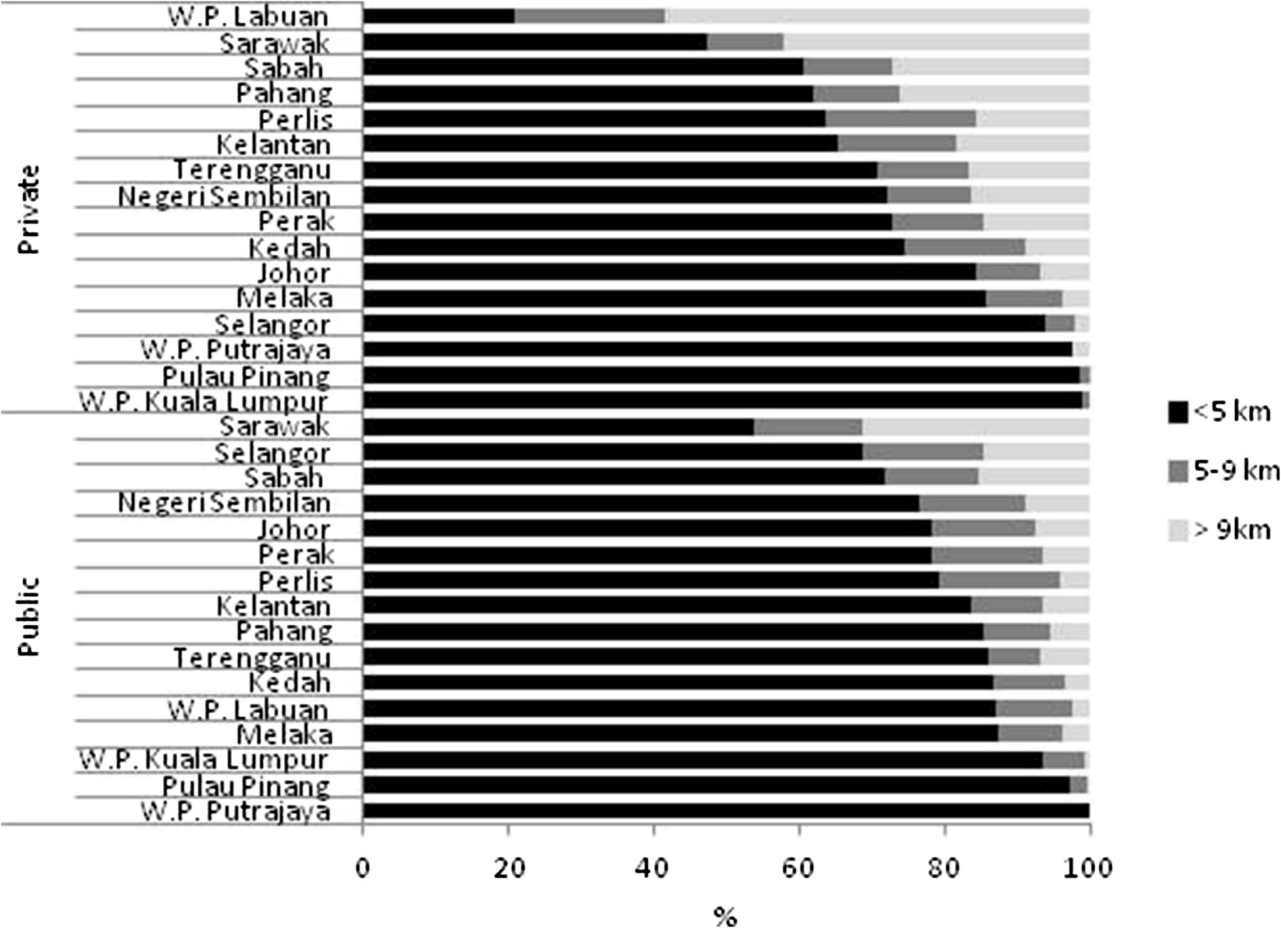
Distance from clinics in Malaysian states

Health professionals identified non-attendance at clinics and non-adherence to medication as a major problem. Transport is important; official data state that 77 % of the population live within 5 km of clinic, and 10–15 % live more than 9 km away from a public or private facility, respectively (Fig. 3) [28]. However, in rural areas, 17 % of the population lives more than 9 km from the nearest public clinic, and 40 % lives as far from a private clinic. In our study, patients in rural sites in both peninsular Malaysia and Sabah identified a lack of affordable transport as a critical barrier to attendance. In Sabah especially, individuals described travelling for up to a day to reach a clinic, walking through jungle and crossing rivers. Many elderly patients rely on their children for transport. In urban areas, patients found difficulty in taking time off work to attend the clinic: *“Some of the private companies don’t accept ‘time-off’, but we [doctors] cannot provide medical leave because [patients] were only here for a few hours”* (HP). Another factor, noted in many settings, is that *“from a patient perspective, hypertension is often a silent disease and patients may not take antihypertensive medications as directed because their positive effects are not as obvious as potential side effects from the medications”* [29]. Interruptions in treatment and drop out were commonly reported; patients stop coming when their BP is under control, to return months later with dangerously high BPs. However, a commonly held view among providers and patients is that many people are in denial about their condition, or, having failed to follow the lifestyle advice given by physicians on diet and exercise, were reluctant to see their doctor. Some providers felt non-adherence is a problem among patients of lower educational status as they believe *“medicine equals money loss”* (HP) whilst the better educated are more adherent so that they remain healthy to work, although they could provide no statistical evidence to support these claims.

A third factor related to the widespread belief that lifestyle modifications were difficult to reconcile with a Malaysian diet high in oil, salt and sugary foods. Key informants were concerned about the affordability of a healthy diet: *“The foods in Malaysia are cheap and varieties which lead to hypertension… lifestyle, stress and workload makes us not active, caused by [the] development of [the] country”* (KI).

Finally, there is no formal process for following up non-attendees in any of the clinics. Although some clinics hold medical records that make this possible, in most clinics patients keep their own records; clinics only become aware of them when they return suffering from complications. The situation is further complicated by patients moving between public and private clinics. A Malaysian information technology system, ‘Tele-Primary Care’, has been implemented in some states, most extensively in Sarawak, that should alert clinic staff when patients are due for their next review and what investigations they require, while informing decisions on their medication. However, it has not been evaluated and nor is it being rolled out nationally.

### Where are patients looked after?

Despite an extensive, heavily subsidised public health care sector, there is a strong and growing private health sector, funded through out-of-pocket payments or company insurance. The public and private facility users differ. Private clinics reported that most of their patients are insured by their employers and that they tended to adhere to their medication “*especially if in the companies’ policy does state that ‘you have to be fit to work with us’*” (HP). Company insurance often allows patients to choose from a number of private clinics, which may disrupt continuity of care (PA).

Those choosing to pay for private care were seeking what was perceived as better quality, with shorter waiting lists and better access to specialists: *“If you had hypertension and heart problems, you will get to see the cardiologists [in a private hospital]; at public hospital you will not necessarily be seen by cardiologist although it’s a cardiology clinic, sometimes only the medical officer in-charge”* (HP).

Other studies in Malaysia have identified *“the choices of medications, the cost to the patients, doctors’ workload, patients’ socioeconomic profile, practice infrastructure, and support facilities”* as playing a role [30]. Yet one doctor argued that “*in terms of diagnosis, full length of investigation, government hospitals are better because we have a strict protocol*” (HP), although this was not supported by the evidence of frequent departures from guidelines. Key informants reported that there is movement between public and private sectors; users with mid-level incomes might use private care for acute illness but move to the public sector care if they develop a chronic condition, require in-patient care or are no longer covered by insurance. Consequently, the public sector was left with the most complicated cases, tending to overwhelm it with patients with advanced disease. However, some of those without insurance and unable to afford private care may still avoid the overcrowded public sector and self medicate or use Traditional and Complementary Medicine (TCM) instead (KI).

#### Traditional and complementary medicine

Use of TCM in Malaysia is believed to be widespread, with *“a significant but unknown proportion of the population consult unregistered traditional medicine practitioners”* [31], although many move between western and traditional systems with the same conditions. A study of the use of TCM for chronic disease management in Ipoh found that *“although 60 % on TCM subjectively felt improvement in BP control, this was not reflected in the actual BP measurement obtained from the medical records”* [32].

Health expenditure data from the Ministry of Health report that 13 % of out-of-pocket expenditures are to TCM providers [27]. A few TCM services, such as Ayurvedic medicine and homeopathy, are provided by the Ministry of Health but most patients pay out-of-pocket for private TCM services and medicines.

Observations and interviews suggested that Ayurvedic practitioners diagnosed hypertension using a sphygmomanometer while those from other traditions diagnosed it on the basis of symptom reports or in patients reporting having been diagnosed by another doctor. There was a divergence of views about the simultaneous use of traditional and western treatments. Whilst it seems acceptable for Ayurvedic medicine to be used alongside western medicine, a Malay traditional medicine practitioner recommended that western medicines should be discontinued when taking herbs. Many patients who report regular use of western medicines for hypertension also mentioned supplemental use of herbal remedies, mainly to relieve perceived side effects of the western medicines. Ayurvedic practitioners also encouraged lifestyle modifications as well as medication, whilst other TCM practitioners focused more on medications. Ayurvedic practitioners were more likely to use registered herbal medications compared with other TCM practitioners.

All three traditional belief systems (Traditional Chinese, Ayurvedic, and Malay) see illness as largely time-limited, reflecting imbalances between factors acting on the body (Appendix Table 6). Consequently, it is difficult to accept life-long treatment for a condition that is asymptomatic, with many TCM practitioners arguing that they can cure hypertension. This view contradicts the need for long-term adherence to medication. One key informant noted that “*more than 50 % do not take their medication regularly. [There are] myths about medication especially among Malays, [that] medication will cause renal failure*”. This concern was common among patients interviewed, believing that long-term daily consumption of any western medication would cause damage to the kidneys, unlike TCM drugs that are *“natural”*. Furthermore some western drugs may not be considered halal, whilst most TCM are considered so, with obvious consequences in a predominantly Muslim majority state.

It was difficult to assess whether advice by alternative practitioners to discontinue western medicines reflected their belief system of illness as a temporary phenomenon or because western medicines were in competition with their products, and thus their income. Some patients in Sabah favoured alternative medication as it was not necessary to follow a strict regimen; others started taking it when they felt that western medication was not working or if they experienced side effects. TCM is seemingly popular with newly diagnosed hypertensive patients, with many *“asking around to get suggestion from their circles to try TCM, mostly herbs”* (HP). Patients will rarely disclose to a medical doctor if they are taking TCM supplements unless complications arise.

In practice, all of these factors seem to play a part in low levels of adherence, especially when combined with long waiting times and the prospect of taking the medication for life.

### Physical resources

There are significant variations in access to equipment and drugs. The facility checklists showed that while health workers in urban clinics reported having no difficulties, those in rural clinics had inadequate equipment of all types, both in terms of supply and quality, with much being very old. Urban clinics reported advising many patients to purchase their own automatic sphygmomanometers, which was not possible in poor rural areas. In the rural sites it was difficult to obtain laboratory results, with samples having to be transported considerable distances.

Key informants at national level reported no problem with access to medicines, contradicting the findings of a 2004 survey reporting widespread non-availability of common medicines [33]. In our study, physicians at urban sites reported few if any problems with access to antihypertensive drugs, whilst those in rural areas reported frequent stock-outs. Although doctors typically prescribed a 3 month supply of drugs, public sector pharmacists typically dispensed it in 1 month or even 2 week instalments to safeguard limited stock (HP), although others said this was to encourage patients to return for regular check-ups.

In the public sector, generic medicines on the national formulary are supplied free to both in- and outpatients. In the private sector, medicine is paid for out-of-pocket, often providing brand-name drugs at higher prices [33]. Patients can purchase anti-hypertensive drugs at any pharmacy with a prescription. Private sector clinics often give 1 month prescriptions so as not to burden their clients financially. None of the patients interviewed reported being unable to access drugs from a public clinic or pharmacy, although some reported going without medication when their prescriptions ran out and they did not have time to obtain more.

Malaysia has, however, a large domestic pharmaceutical manufacturing sector, with “*80 % of drugs being locally manufactured*” (KI), enabling some cost savings in recent years.

### Human resources

The Malaysian health workforce faces shortages of health workers, with 0.9 physicians per 1000 population, which is below the 2.2 per 1000 average in upper middle income-countries. Although the public sector is well resourced, only 7 % of doctors work in primary health care. While the FMS, with their additional skills in family medicine, can provide additional support, in 2008 there were only 149 in the whole country [34]. Our study indicates more pronounced shortages in rural areas, with rural physicians described feeling severely overworked due to staff shortages. The rarity of visits by FMS was also regarded by staff and patients as contributing to long waiting times. The Ministry of Health determines staff allocation according to clinic catchment areas, so some will be allocated a full-time FMS, whilst others with fewer than 250 patients per day will receive only intermittent visits from a FMS, with most care provided by Medical Officers with basic training.

Continuity of care was of concern at all public sector sites, with patients seeing a different doctor each time they attend the clinic. This posed a problem given the significant variations in hypertension management. This is exacerbated by the high turnover of physicians and medical assistants in many areas, with the Ministry of Health offering only short contracts and arranging frequent redeployments in rural areas. Undergraduate medical training does not focus on primary care, so that newly qualified doctors are often unfamiliar with the conditions seen at this level.

Some clinics did, however, describe implementing appointment booking systems so that Medical Officers can see the same patients and thus maintain continuity of care. Both doctors and patients regarded this as important in building trust and rapport, with benefits for adherence to treatment.

The involvement of other health professionals is an area where rhetoric departs from reality, as key informants described the involvement of dieticians and nutritionists in hypertension management, supporting patients with lifestyle modifications, but these cadres were not found in any of the clinics observed. Furthermore, at all sites, health professionals reported a language barrier between Malay staff and Chinese and Indian patients, making communication difficult; this was further complicated given the indigenous languages in rural Sabah.

### Intellectual resources

#### Guidelines

As noted above, clinical guidelines for management of hypertension exist and are widely disseminated. Development of Clinical Practice Guidelines (CPGs) is undertaken by working groups under the auspices of the Ministry of Health, and revised every 5 years to account for newly emerging evidence. The CPGs are published in various formats for health professionals in work and in training, and for patients, and appear on the Ministry of Health website. All health professionals interviewed reported using them, albeit tempered by their clinical judgment, finding them straightforward and clear. Some health professionals, however reported being too “*overwhelmed with patients to follow everything inside the CPG*” (KI). Given the evidence that guideline adherence improves hypertension control [35] this could have implications for quality of care. There was also concern that guidelines for different conditions were sometimes inconsistent. Private clinics are even less regulated and do not have *“SOP [standard operating procedure] for their medical officers; it’s up to the individual judgment”* (HP).

#### Patient awareness of hypertension

There was widespread concern among health professionals about a poor understanding of hypertension and, specifically, the need to maintain treatment when asymptomatic, as noted above. Educational materials produced by the Ministry of Health came in for particular criticism from health professionals. These were developed with little input from target audiences such as patients. They took no account of the deeply entrenched health beliefs, were considered to have little impact on raising awareness of hypertension or its risk factors, and were characterized by paternalistic messages delivered through a top-down approach. One key informant reported that the education materials were: *“Not effective at all, we are not getting the message across…I think the education materials that we have now are not dynamic, not customized. It’s too general”*.

Few patients reported receiving any leaflets or other materials from the clinics. Little printed information was visible in any of the clinics, and what was in them was outdated and poor quality. In Tanjung Sepat, however, health professionals had developed their own hypertension information pamphlets but none were found in the clinics; a nurse explained that parents would often give them to children in the waiting area to play with and they had not been replaced. The materials are produced by Ministry of Health so clinics must request them from the Ministry, and these are therefore often lacking in the clinic sites. As a consequence, patients reported that the information they did obtain was mainly from the media and other community members; many cited television advertisements by the Ministry of Health. The increased use of the internet has not necessarily promoted access to evidence-based information. There was particular concern that the mass media was redolent with advertisements for unhealthy products, such as certain foods and products or health supplements, as well as advertisements for ‘wonder-drugs’.

### Social resources

Most patients reported having been offered counselling on how to manage their hypertension by their physicians. The Ministry of Health is *“specifically looking at patient support group or peer support groups”* (KI), but other informants reported that the *“ministry have failed to empower other agencies to [be involved/volunteer in hypertension programmes] because the ministry is thinking that we are the better person with this”.* A peer support group had been established in the Tanjung Sepat clinic but one health professional there noted: *“Peer support groups… I find it still lacking in Malaysia… maybe because of our setup our cultural belief that not many patients are willing to actually share their experience”*.

There is also a Hypertension Club in Kuala Lumpur that allows patients to exchange information, although no patients interviewed had attended this. There are no specific state benefits for people suffering from NCDs.

The national strategy on NCDs [36] proposes support for health promoting initiatives, such as those in schools, although some key informants considered that these lacked popular support and had achieved limited success. There is limited social support for patients: the strategy requires that health clinics be linked to the Social Welfare Agency, to support patients who need specific support or home-visits, but patients reported that applications for support take a long time.

## Discussion

Innovations in health care delivery are frequently implemented without any attempt to evaluate them and assess the extent to which they respond to patient needs. Even when they are subject to an evaluation, it is rare for them to be tailored to the local context [37]. This is in marked contrast to the development of medicines, where vast sums are spent to develop and refine the product, in terms of dosage, presentation, and mode of administration before it ever gets near a patient. One review of malaria programmes concluded that many “*fail to utilize findings from social science research and as a result, achieve minimal results*” [38].

This study has provided valuable insights into the management of hypertension in Malaysia. It finds that, while NCDs are now high on the political agenda, the focus is predominantly on diabetes, with rather less attention to other conditions such as hypertension. Patients with hypertension are diagnosed when they present with symptoms, which may or may not relate to their hypertension but this suggests that many may be diagnosed at a relatively late stage. Mass screening activities take place but, although not subject to formal evaluation, seem to be of limited value as few people in whom elevated blood pressure is detected are followed up. Once commenced on treatment, arrangements are made for routine follow up but attendance is low, largely because of a combination of travel times (rural areas), difficulty taking time off work (urban areas), and long waits in clinics. The problems are greatest in rural areas, which face shortages of basic equipment and medicines, with shortages of physicians who change regularly, making continuity of care difficult. In more remote areas where many patients are from indigenous groups there may also be language barriers. However, even in urban areas, many of those who can (mostly those with insurance cover), seek private care. Guidelines exist and are generally viewed positively, although some are contradictory and there may be insufficient time to adhere to them in detail. Patient adherence to treatment seems low and, at least in part, this seems to reflect traditional beliefs, from different sources, that view disease as a transient imbalance that, once corrected, does not require continuing treatment. Linked to this, it is apparent that many people seek traditional remedies, in combination with or instead of recommended evidence-based treatments. Notwithstanding these concerns, there does seem to have been some progress since a 2007 evaluation of hypertension services [39].

A recent study undertaken in a tribal area of India echoed our findings in Malaysia, such as reliance on traditional medicine, non-adherence to medication, inappropriate education materials, and logistic challenges in accessing care [40]. Even though that study was undertaken in a completely different setting, its findings are remarkably similar to ours, indicating a broader relevance.

Before discussing the implications for design of an intervention, it is necessary to reflect briefly on the strengths and limitations of this innovative approach to appraise the health system. Its main advantage is that it takes user perspective and identifies upstream bottlenecks at local, district and national level, drawing on theories and frameworks for analysing health systems operations. It uses a range of qualitative and quantitative methods in an iterative and reflexive approach allowing for collection and analysis of data that is rigorous yet pragmatic. Yet whilst a wide range of stakeholders were enrolled in this study, the sampling methodology created an inevitable bias towards those patients who attend health clinics, as well as towards providers and key informants who were willing to provide information, which tended to emphasise the role of the public sector. There was also a bias towards the Malay population due to language spoken by the researchers. However, we did triangulate our findings with different sources, including existing literature. Thus, as in an earlier study, we found that patients most likely to access hypertension care in the public sector are older, female and of Malay ethnicity [30].

Our findings have been very important in designing an intervention that will now be evaluated as a potential means of improved hypertension management. Table 3 demonstrates the various barriers to effective hypertension management within and outside the health system in Malaysia, and at different levels that must be tackled in the design of an intervention. The most important barriers relate to the management and clinical practice within clinics and health beliefs. Both have implications for adherence to treatment, although it is important to note that poor adherence is a virtually universal problem, even if the precise causes vary [41–43]. We found little evidence for any major financial barriers to accessing care or medication, since public sector care is heavily subsidized and medication is widely available in the public and private sectors. However the rapid staff turnover and long waiting times are a matter of concern. Additionally, the intervention will need to tackle particular problems in rural areas, and among different population, ethnic and socio-economic groups. This is a very different situation from that in Colombia, where this study is also being undertaken, where shortages of drugs are widespread everywhere [44]. Nor are there major concerns about financial barriers, with out-of-pocket payments only being paid by those opting for private care, most of whom are at least partially covered by occupational health insurance. We did not find evidence of informal payments, unlike the situation in many other middle income countries. Most clinics reported having adequate diagnostic equipment, even if not always the most modern, but there were significant managerial problems.

**Table 3:** Barriers to hypertension control in Malaysia

	Community and household level	Health service delivery level	Health sector policy and governance level	Environmental and contextual characteristics
Hypertension clinical management	- Health beliefs leading to non-adherence to drugs	- Lack of routine screening	- Lack of effective public awareness campaigns	- Lack of understanding of access issues from a patient perspective
	- Supplementary use of traditional therapies, sometimes leading to discontinuation of Western medication	- Lack of follow-up from community outreach activities	- Lack of incentives for health staff or clinics to improve quality of management or care	- Weak response to risk factors for hypertension, such as food industry regulation
	- Patients seeking care across public, private and TCM providers, disrupting continuity of care	- Little time with patients for effective counselling and management	- Lack of continuity of care between and within public and private sector	- Lack of regulation of TCM
		- Failure to dispense enough medications to last between check-ups		- Lack of evidence for TCM
		- Overburdened public health system puts pressure on resources due to private sector dumping		
		- Clinics are not open out of working hours		
External issues	- Access to clinics due to transport or working hours		- Lack of social support or counselling available for hypertension patients	- Weak civil society
	- High fat and oil diet is cheap and accessible			- Lack of regulation of food and tobacco industry

Source: Authors’ assessments, using a structure adapted from Hanson et al., [53]

This analysis shows how, in Malaysia, an intervention must include messages that take full account of deeply held beliefs that do not recognize long term asymptomatic conditions and lead many patients to use traditional medicines that may conflict with western approaches to management. There is growing recognition of the need to understand health beliefs in influencing medication adherence, with a recent systematic review showing both similarities in many diverse settings worldwide but also some cultural specificities [45]. This paper adds to that literature which, so far, has provided evidence mainly from high income countries. As elsewhere, there is much scope to increase understanding of hypertension among both patients and health professionals, with evidence from other settings that patient empowerment and education can improve health outcomes in hypertensive patients [46] when done well. However there is a need to test messages related to the risks of hypertension and the need for long-term treatment even when asymptomatic. This requires skills in cognitive psychology and market research. There is now considerable experience with social marketing in some areas of health promotion, for example, for reproductive health [47], but less so in relation to cardiovascular disease. However, where it has been tried, it has led to innovative methods, such as the use of community workers or house parties to raise awareness [48]. It is, however, important that these approaches do not become simply tokenistic in their efforts to address cultural diversity [49]. It is also important that the messages emerging should be sustained and consistent throughout the health system, which will require harmonization of guidelines and training for health workers to work towards the same goals [50].

The analysis also identifies the high opportunity cost to patients imposed by transport problems and the time required to obtain care. The former is difficult to tackle by actions within the health system but both may be alleviated to some extent by extending clinic hours. Given the costs that arise in these ways, there may also be a case for considering conditional cash transfers as incentives to attend regular follow up. These have been shown to be effective in relation to other aspects of health care [51], but there are considerable financial and governance challenges.

There is also a clear need to improve detection and treatment of hypertension. Ideally, every patient over a certain age would have their blood pressure measured at every encounter with a health worker. However, this will not be easy (or indeed possible) with the staffing levels available. Even for patients who have had treatment initiated, staffing levels are inadequate, reflecting a national shortage of physicians and maldistribution, with very few working in primary care, especially in rural areas, where those that do exist are employed on short-term contracts or are frequently redeployed. Malaysia also has a low density of nurses compared to its neighbours, although the situation is complicated because it is both a major importer and exporter of nursing staff [52]. This points to the need for task-shifting, and for more of the workload to be undertaken by mid-level non-physician health workers. However, this must be accompanied by enhanced training of those such as nurses, who could assume new roles. This will also require an imaginative response to the legal prohibition on cadres other than doctors prescribing medication. One possibility might be to allow nurses to vary dosages within protocols although, in the longer term, legislative change will be needed.

It will be important to consider new approaches, such as those made possible by technological advances. For example, non-physician health workers could screen patients using electronic sphygmomanometers, complete a check list on a smart phone or tablet that would then calculate cardiovascular risk scores (and stratify individuals into high, medium and low risk), identify concomitant risk factors that require modifications (e.g. smoking or diabetes), key contraindications to specific drugs (e.g. a beta-blocker if there is asthma) and recommend the use of low dose single pill combination medicines, which will reduce the number of “steps”, visits and drugs for the health care provider and the patient and generate specific “warnings” when the patient needs to be referred “upwards” to a physician. Such a system can increase adherence to clinical practice guidelines, provide documentation and quality control, minimize costs, and enhance the effectiveness of the non-physician health worker. This approach can also provide answers to frequently asked questions to enable the non-physician health workers to dispel common myths and misunderstandings about hypertension and is being piloted in the next phase of the HOPE 4 study in both Malaysia and in Colombia. However, in the longer term, there is also a need to ensure that there are sufficient staff available and that the country develops measures to increase training and retention.

## Conclusion

This appraisal of the Malaysian health system has revealed how, despite a highly-subsidized and mostly easily accessible public health system with good availability of drugs, there are still significant barriers to effective hypertension management. These lie mainly in a limited popular awareness of hypertension as a chronic yet manageable condition, competing health belief models, a shortage of physicians, and poor management of available resources. Although these factors can be found in many settings, an understanding of their specific nature is important for the development of a contextually appropriate intervention. Yet, too often, this step is missing in health systems research. Integrated and innovative solutions are needed to manage patients using non-physician health workers and, if successful, can overcome many of the barriers to effective hypertension control.

## Appendices

**Table 4:** Key characteristics of the Malaysian health system

The Malaysian health system comprises a mix of public and private funding and provision. The centrally organized public health sector provides 82 % of inpatient care and 35 % of ambulatory care, with the rest provided through the fast-growing private sector [31]. The public sector comprises a large network of primary care clinics, each typically serving a population of 20,000, staffed by a doctor (Medical Officer), pharmacist and nursing staff and overseeing a number of nurse-staffed community clinics providing maternal and child health. Doctors in public clinics are supported by Family Medicine Specialists (FMS), who visit regularly to advise on patients with more complex conditions. Secondary care is provided in five types of hospitals (defined in terms of size, specialization, and whether specialists are resident or visit). There is no gate-keeping system, with Ministry of Health having an “open door policy on outpatient services and hospital admissions” [31], allow patients to choose where they seek care. Public health services are administered centrally by the Ministry of Health (Ministry of Health), although the Ministry of Higher Education also runs university teaching hospitals, the Ministry of Defence has military health services, and, in some remote areas, the Ministry of Aboriginal Affairs provides services for indigenous populations.
As well as the formal medical system, there are also many traditional, complementary and alternative medicine (TCM) practitioners drawing on the beliefs of the three main communities: traditional Malay healers (bomohs), Traditional Chinese Medicine practitioners, and Ayurvedic practitioners serving the Tamil population.
*Financing and insurance:* Total health expenditure in 2011 was 4.26 % of GDP, equivalent to US$422 per capita [54], which is low compared with neighbouring SE Asian countries [55] and upper-middle income countries overall. In 2011, 45 % of total health expenditure was from Ministry of Health, with 37.7 % coming from out-of-pocket payments (OOP); OOP payments account for 78 % of private sector funding. Private insurance contributed only 7 % of Total Health Expenditure [54]; and Social Security Organisations account for only 0.68 %. Public clinics receive a fixed annual budget, linked to performance indicators and budget lines, with the rationale of providing equitable levels of care across the country [31]. Primary care is largely subsidized by Ministry of Health as part of their efforts towards universal health care coverage, brought in in the 1980s. Attendances at primary care clinics involve a small co-payment of RM1 (€0.25) for registration and regular consultation, and RM5 (€1.25) for specialist consultations. With the large subsidized public sector, there is no government social insurance scheme. The private sector has expanded rapidly in recent years, primarily in urban areas where investment in new public facilities has not kept pace with population growth and where disposable incomes are higher. There is a small private insurance market, primarily for expensive hospital treatment.

**Table 5:** NCDs in Malaysia

NCDs have been on the policy agenda for almost two decades after a substantial rise in their prevalence was noted in the National Health and Morbidity Survey II (NHMS II, 1996). In response, the National Diabetes Prevention and Control Programme was launched in 1996 and strengthened in 2000 [56]. It was the precursor of the National Strategic Plan on Non-Communicable Diseases (NSP-NCD) 2010–2014, [36] which sets out a comprehensive action plan for the major NCDs, setting a new direction.
This new political will was seen as having driven certain structural changes, such as the establishment of an NCD section within the Disease Control and the Family Health Development Divisions of the Ministry of Health, with complementary actions in other ministries and greater engagement by the Malaysian government with NCDs in the global arena. However, there are also concerns that the Ministry of Health is still dominated by silo working that hindered progress, with others expressing concern that the government’s programmes remained focused on individual diseases. It was suggested that this might be due to an unwillingness to confront powerful vested interests in the food and tobacco industries.
A high priority has been given to diabetes, which has been supported by a strong lobby since the 1990s. A 1996 Healthy Lifestyle campaign on Diabetes is cited as an example of positive action [31].

**Table 6:** Complementary and alternative approaches to hypertension in Malaysia

Traditional shamanistic Malay healers (bomohs) view illness as produced either by physical factors, such as temperature, foods, small particles (“kuman”), or wind (“angin”), which may either act directly or under the influence of spirits, and by the spirits themselves, which may reside within individuals or may have rendered individuals or animals with whom they have contact toxic (“bias”) [57] . Individual bomohs are associated with particular conditions, causes and remedies, which can include incantations, trances, herbal remedies and many other modalities [58]. Other traditional Malay practitioners use herbal remedies, some of which are now manufactured commercially by producers adhering to principles of Good Manufacturing Practice. Yet others use physical therapies, such as massage, often accompanied by prayers.
Traditional Chinese Medicine is based on three concepts. These are vital forces, flowing through 12 channels in the body, Yin-Yang, where phenomena such as heat and cold are dependent but in opposition to one another, and Wu-Hsing, in which 5 elements are associated with body organs, such as fire and the heart. It views hypertension as being due to excessive blood in the body, with dizziness and headache caused by the blood rising to the head. This gives rise to treatment by bloodletting through small cuts at the back of the scalp. The excessive blood is attributed to excess body heat. This can be treated by avoidance of certain “hot” foods, such as red meat and durian, and consumption of “cold” ones, such as starfruit. It may also be treated by exercise regimes [59] or herbs [60].
Ayurvedic medicine recognises three forces, or “dasha”, each with characteristics derived from space, air, fire, water and earth. These are Vata, Pitta, and Kapha. Hypertension is due to a derangement of Vata and, in some cases, Pitta. Treatment is by herbs, including Rauwolfia, the source of the early anti-hypertensive reserpine, and others [61], as well as yoga and exercises.
